# Irisin in Blood Increases Transiently after Single Sessions of Intense Endurance Exercise and Heavy Strength Training

**DOI:** 10.1371/journal.pone.0121367

**Published:** 2015-03-17

**Authors:** Håvard Nygaard, Gunnar Slettaløkken, Geir Vegge, Ivana Hollan, Jon Elling Whist, Tor Strand, Bent R. Rønnestad, Stian Ellefsen

**Affiliations:** 1 Lillehammer University College, Lillehammer, Norway; 2 Hospital for Rheumatic Diseases, Lillehammer, Norway; 3 Innlandet Hospital Trust, Lillehammer, Norway; Charité-Universitätsmedizin Berlin, Campus Benjamin Franklin, GERMANY

## Abstract

**Purpose:**

Irisin is a recently identified exercise-induced hormone that increases energy expenditure, at least in rodents. The main purpose of this study was to test the hypothesis that Irisin increases acutely in blood after singular sessions of intense endurance exercise (END) and heavy strength training (STR). Secondary, we wanted to explore the relationship between body composition and exercise-induced effects on irisin, and the effect of END and STR on muscular expression of the irisin gene FNDC5.

**Methods:**

Nine moderately trained healthy subjects performed three test days using a randomized and standardized crossover design: one day with 60 minutes of END, one day with 60 minutes of STR, and one day without exercise (CON). Venous blood was sampled over a period of 24h on the exercise days.

**Results:**

Both END and STR led to transient increases in irisin concentrations in blood, peaking immediately after END and one hour after STR, before gradually returning to baseline. Irisin responses to STR, but not END, showed a consistently strong negative correlation with proportions of lean body mass. Neither END nor STR affected expression of FNDC5, measured 4h after training sessions, though both protocols led to pronounced increases in PGC-1α expression, which is involved in transcriptional control of FNDC5.

**Conclusion:**

The results strongly suggest that single sessions of intense endurance exercise and heavy strength training lead to transient increases in irisin concentrations in blood. This was not accompanied by increased FNDC5 expression, measured 4h post-exercise. The results suggest that irisin responses to resistance exercise are higher in individuals with lower proportions of lean body mass.

## Introduction

Metabolic diseases such as obesity and diabetes are undoubtedly some of the most challenging health issues of our times [1]. It is hence important to identify simple and inexpensive methods to counteract them, with simultaneous elucidation of underlying causalities. In 2012, Boström et al. [2] reported the finding of an exercise-inducible hormone, irisin. They showed that irisin mediates increased energy expenditure in mice resulting in improved weight regulation and glucose homeostasis, and suggested a role for it in humans [2]. This makes irisin a potential and intriguing target for preventing and treating obesity and metabolic diseases [3]. However, the relevance of irisin in human physiology is not fully understood [4], with effects of endurance and in particular strength training remaining obscure. There is need for more knowledge before we can pursue it as a potential therapeutic tool.

Boström et al. [2] found irisin to be cleaved from the membrane protein FNDC5 in muscle and released into the circulation. This was found to be under control of the transcriptional coactivator peroxisome proliferator-activated receptor-γ coactivator-1 α (PGC-1α)[2]. In general, PGC-1α mediates many of the physiological adaptations associated with endurance training [5], and conveys many of the known health-related physical adaptations, such as mitochondrial biogenesis [6]. PGC-1α comes in different isoforms [7]. Whereas, PGC-1α splice 1 is considered the traditional isoform of the gene, being involved in endurance-related adaptations in skeletal muscle, the recently identified PGC-1α4 seems to be predominantly involved in adaptations to resistance training [8].

Originally, Puigserver et al. [9] identified PGC-1α as a regulator of thermogenesis in skeletal muscle and beige adipose tissue in mice via its activation of mitochondrial genes such as uncoupling protein 1 (UCP-1), which is specific for brown fat. UCP-1 uncouples proton currents across the inner mitochondrial membrane from ATP production, allowing heat production [10]. In this manner, UCP-1 provides beige adipose tissue with the ability to oxidize fat and glucose in excess of energy demands, giving it a potential role in controlling body mass [10, 11]. In accordance with this, the increased energy expenditure and improved glucose homeostasis mediated by irisin in mice occurs in a UCP-1-dependent manner [2], independently of physical activity and dietary habits. In essence, irisin converts white adipose tissue into beige adipose tissue [2], and may thus constitute the missing link between PGC-1α and its positive effects on systemic health, ranging from controlling fat mass, impairing muscle wasting, increasing bone mineral density, preventing insulin resistance, and reducing systemic inflammation [12]. A potential role of irisin in health and longevity is supported by several cross-sectional studies [13–19], though its physiological role in humans is debated [4].

The training-induced nature of irisin remains uncertain in humans. While some studies have found irisin concentrations to increase in blood immediately after singular bouts of exercise [20–25], others have failed to do so [26, 27]. Inconsistencies also exist with regard to effects of training on expression of FNDC5 mRNA. While FNDC5 expression originally was claimed to increase after acute exercise [2], more recent studies have reported no or only minor increases [26, 28]. So far, few studies have explored the acute effect of strength training on irisin. Pekkala et al. [26] found no effect on irisin after a heavy strength training session, being restricted to exercises of the legs. In a long term study we recently identified a surprising negative correlation between changes in rested-state irisin levels following 12 weeks of strength training and lean body mass [29], suggesting a complex interrelationship between irisin and strength training.

The purpose of this study was to test the hypothesis that irisin levels in blood increase acutely after single sessions of both END and STR. Secondary, we wanted to explore the relationship between body composition and exercise induced effects on irisin, and the effect of END and STR on the muscular expression of FNDC5.

## Materials and Methods

### Subjects

Nine healthy individuals (two women and seven men) participated in the study. Subject characteristics are shown in table 1. All subjects were classified as moderately trained, having performed 1–5 sessions of endurance and/or strength training per week for the three months preceding study enrolment, including at least two endurance training sessions and two strength training sessions per month.

**Table 1:** Subject characteristics.

Age	Height	Weight	BMI	lean body mass	Body fat content	VO2max
32±9 years	180±10 cm	80±11 kg	24,5±2,4Kg/m^2^	61±10 kg	24±6%	50±7 ml/kg/min

Mean ± SD.

### Ethics Statement

The Regional Ethics Committee (REK Sør-Øst, Norway) approved the study, and all subjects gave their written informed consent.

### General design

**Fig 1:**
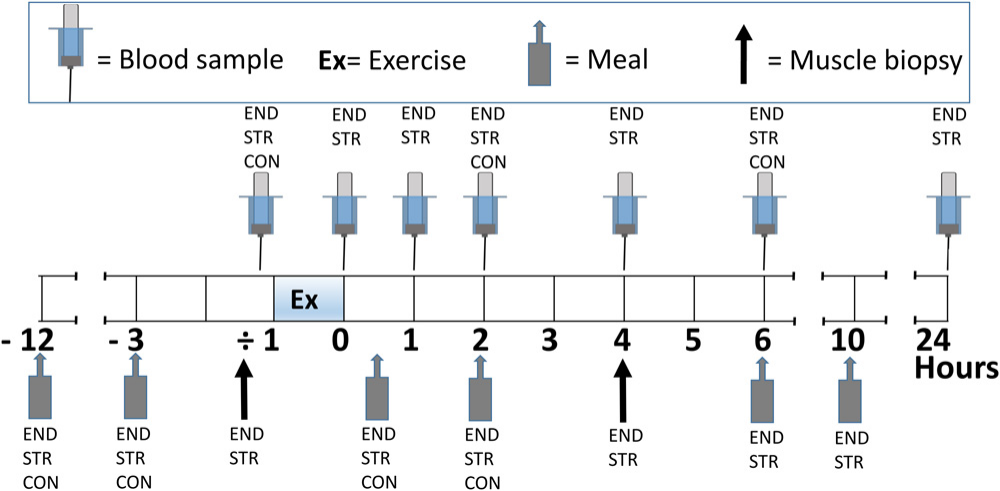
Schematic presentation of the protocol of the test days. The time line spans the period from the evening prior to exercise days (-12) to the subsequent morning to exercise days (24 hours subsequent to exercise). 0h = the end of the 60 minutes exercise sessions. There were three test days; one day with a session of intense endurance exercise (END), one day with a session of heavy strength training (STR) and one day without exercise (CON). CON was similarly structured as END and STR, except that no exercise was performed, no muscle biopsies were sampled, and blood samples were only sampled at-1h, 2h and 6h.

The study was performed using a randomized crossover design. Each subject carried out three experiments on three separate days, separated by at least six days and no more than 22 days between each: one test day with a session of intense endurance training (END), one with a session of heavy strength training (STR), and one without physical exercise (CON). All exercise sessions started at 08.00 a.m. and lasted for 60 minutes. Subjects were located to indoor accommodations with stable temperatures throughout all experiments and were sedentary except for during exercise sessions. Blood samples and muscle biopsies were collected at standardized time points. All experimental days were identical (Fig. 1), except for the following: I) they involved different exercise regimes, II) on the day of CON, blood sampling was limited to fewer time points and III) on the day of CON, no muscle biopsies were sampled. To ensure maximal effort during END and STR, subjects were orally encouraged.

Subjects were instructed to refrain from physical exercise for the three days preceding experimental days, and were instructed to standardize their diet for each of the three-day periods prior to each test. On the evening before each test day, a meal was ingested that contained >50g carbohydrate. The breakfast before tests also contained >50g carbohydrate. On test days, the research team provided subjects with all food. On the first of the test days, each meal consisted of self-chosen amounts of food ingested at standardized time points (Fig. 1). Food intake was carefully registered, and dietary standardization was achieved by repeating this food intake on test days two and three. Mean intake on each test day (breakfast to evening meal) was as follows (±SD): 13719 ± 2593kJ, 22 ± 3 E% protein, 35 ± 5 E% fat, 43 ± 6 E% carbohydrate. Notably, one subject vomited after STR and one subject vomited after END (both due to exhaustion), ensuring vomiting-balance between protocols.

### Exercise

END: The endurance exercise was performed as one session of intermittent high intensity treadmill running on self-selected speed and 5% inclination. After a 20 min warm-up at low intensity, subjects underwent 6 X 5 min work intervals, separated by two minutes of rest between each interval. At the end of each work interval rate of perceived exertion was aimed to reach ≥18 on the Borg 6–20 scale [30]. The aim of the entire endurance session was to achieve the highest possible average velocity, with the last interval being an all-out effort. Mean blood lactate concentration immediately after the last interval was 10.1 ± 2.8 (SD) mmol/L (Biosen C-line, EKF-diagnostic GmbH, Germany). VO_2max_ was also sampled during the last interval (Oxycon Pro, Erich Jaeger, Hoechberg, Germany) for subject characteristics (Table 1).

STR: At the start of their strength training session, subjects performed 5-min general warm-up on a stationary cycle ergometer at self-selected moderate intensity. Thereafter, one set of specific warm-up preceded each particular strength exercise, except for half squats, for which two sets of warm-up were performed. Exercises were as follows (listed in chronological order): half squat in a smith machine, leg press, leg curl, seated chest press, seated rowing, shoulder press, latissimus pull-down and biceps-curl. Every exercise was performed as three sets of 10–12 repetitions maximum (RM), with two minute rest periods between sets and exercises. All STR sessions was supervised by an investigator. Subjects accomplished 10.5 ± 0.8 (SD) and 10.0 ± 1.0 (SD) repetitions before exhaustion in the lower and upper body exercises, respectively.

### Sampling and analysis

Blood samples were drawn from an antecubital vein 15 min prior to exercise, immediately post-exercise, and thereafter 1h, 2h, 4h, 6h and 24h post-exercise (END, STR) (Fig. 1). On the control day, blood samples were limited to the time points corresponding to 15 min pre exercise and 2h and 6h post-exercise. Blood was drawn into EDTA tubes, centrifuged immediately at 2600g for 12 minutes and plasma was stored at -80°c until analysis. Plasma irisin was quantified using commercially available ELISA kits (EK-067–29, Irisin Recombinant, Phoenix Pharmaceuticals, Inc, Burlingame, USA). All samples from a particular subject were analyzed using the same plate (intra-assay). Intra- and inter-assay coefficients of variation were 3.5% and 16.7%, respectively.

Muscle biopsies were sampled 30 min prior to END and STR and 4h post-exercise (Fig. 1). Following non-invasive local anaesthetization (Emla cream 2,5% lidocain/ prilocain, AstraZeneca, Karlskoga, Sweden, was applied on the skin), muscle biopsies were collected using a microbiopsy system (Bard Magnum, Reusable Core *Biopsy* System, Bard, NJ, USA) with 14 gauge needles. Biopsies were sampled from *musculus* vastus lateralis, at approximately 1/3 of the distance from the lateral side of the patella to the anterior spina iliaca superior. Biopsies were immersed immediately in RNAlater (Ambion, Life technologies, Carlsbad, CA, USA) and treated according to manufacturer’s protocol, before storage at -80°C until RNA extraction. Total RNA was extracted from muscle biopsies using TRIzol reagent (Invitrogen, Life technologies, Carlsbad, CA, USA), as previously described [31], whereupon reverse transcription was performed on 500 μg total RNA using Superscript III Reverse Transcriptase (Invitrogen, Life technologies, Carlsbad, CA, USA), primed with both random hexamers (Ambion) and oligo d(T) (Ambion), according to manufacturer’s protocol. Expression of FNDC5, PGC1α-splice 1 and PGC1α-splice 4 was determined by quantitative real-time RT-PCR (qRT-PCR), using SYBR Select Master Mix (Invitrogen, Life technologies, Carlsbad, CA, USA) and the 7500 Fast Real-Time PCR System (Applied Biosystems, Life technologies, Carlsbad, CA, USA), employing protocols and primers as previously described [29, 31]. For each qRT-PCR reaction, cycle threshold (Ct) was calculated using the 7500 Fast Real-Time PCR System software in an automated manner and priming efficiencies (E) were calculated using the LinRegPCR software [32, 33]). For final calculations of target gene expression, we utilized average priming efficiencies, calculated separately for each primer pair. Target gene expression was determined using GeNorm [34]. GeNorm-based analyses were based on geometric evaluation of the expression of five frequently utilized reference genes: peptidylprolyl isomerase A (PPIA, cyclophilin A), β_2_-microglobulin (β_2_m), ribosomal protein L32 (RPL32), β-actin (β-a), and polymerase (RNA) II (DNA directed) polypeptide A (PolR2A). PPIA and β_2_m were evaluated to be the two most stable reference genes, with M-values well below 0.7, the limit set by Vandesompele et al. [34], and were utilized for final calculations of normalization factors. Both PGC1α-splice 1 and PGC1α-splice 4 were included in our analyses, due to their known association with endurance training and strength training, respectively [5, 8].

### Body composition

Body composition was determined after the intervention was completed by dual-energy x-ray absorptiometry [35] using a Lunar Prodigy densitometer (Prodigy Advance PA+302047, Lunar, San Francisco, USA), performed in a highly standardized manner. Subjects refrained from training for the 24 hours preceding the measurement, and avoided ingestion of food or liquid for the 3 hours preceding the measurement. Lean body mass (LBM) was calculated by subtracting body fat mass and bone mineral content from total body mass.

### Statistics

Data were tested for normality and analyzed with IBM SPSS statistics, version 20.0. Main effects of the exercise interventions and time after exercise on irisin concentration were analyzed with a general linear model for repeated measures. Pairwise differences between corresponding mean values of the experiments, changes from baseline within each experiment, differences in time to peak irisin concentration between END and STR and changes in gene expression were assessed using paired t tests. Correlations were estimated with Pearson`s method, except when normal distributions were disturbed, whereupon Spearman`s method was used. The α-level was set to 0.05. For statistically significant correlations (p<0.05), correlation coefficients (r) were interpreted according to Hopkins et al. [36]: r<0.1 = trivial, 0.1–0.3 = small, 0.3–0.5 = moderate, 0.5–0.7 = large, 0.7–0.9 = very large, 0.9 = nearly perfect, and 1.0 = perfect. Data are presented as means ± SEM unless otherwise stated. Figures were produced using SigmaPlot 12.0, Systat Software Inc. and IBM SPSS statistics, version 20.0.

## Results

When comparing main effects of singular sessions of END and STR on plasma irisin there was an effect of time after exercise (repeated measures; F = 5.873, p = 0.007). There was no difference between the two types of exercise, but a tendency towards an interaction between type of exercise and time (F = 2.999, p = 0.059). When the three time points of CON were compared with corresponding time points on END and STR, there was an effect of intervention (repeated measures; F = 4.910, p = 0.022).

**Fig 2:**
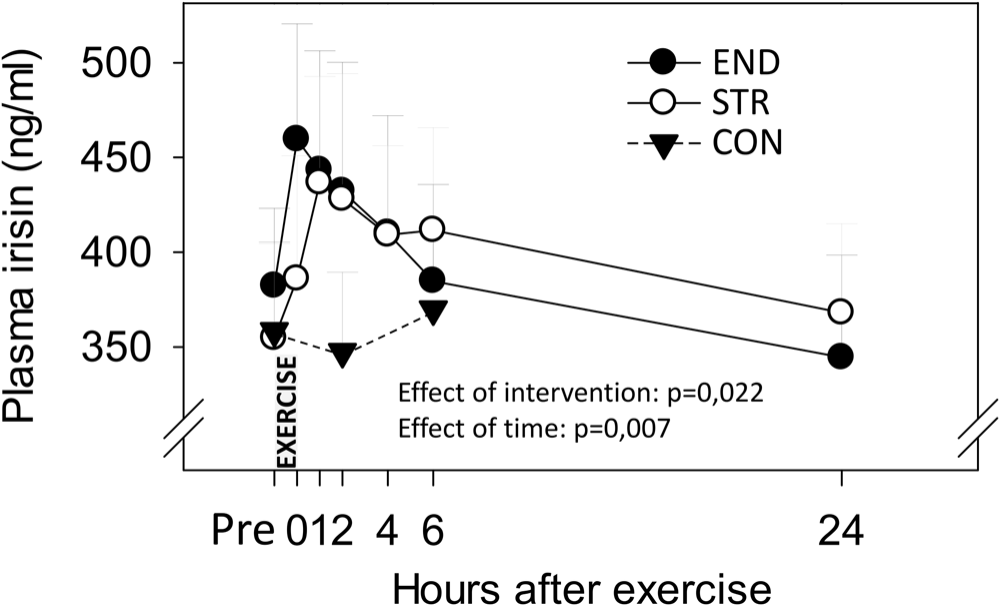
Plasma concentration of irisin. Plasma concentration of irisin measured following singular sessions of intense endurance (END) or heavy strength training (STR), or on a control day (CON) without training. Data are mean values ± SEM. The type of intervention had a significant effect on plasma irisin levels (p = 0.022; based on analyses of values from pre, 2h and 6h). There was also an effect of time after exercise (p = 0.007).

Baseline (pre) results for plasma irisin concentrations were 358±47, 382±41 and 355±50 ng/ml for CON, END and STR, respectively, and did not differ between test days (Fig. 2). On the day of CON, no change was seen in plasma irisin levels from pre to the other sampling points (Fig. 2). Following END, plasma irisin increased to 459 ± 61 ng/ml at 0h (p = 0.037) and to 443 ± 63 ng/ml at 1h (p = 0.05). At 2h post-exercise, irisin levels were 432 ± 68 ng/ml, which was not different from pre values, but higher than the corresponding value on CON (347 ± 42 ng/ml; p = 0.04). Thereafter irisin values decreased, until 24h. Following STR, plasma irisin increased to 437 ± 56 ng/ml at 1h (p<0.001) and tended to increase to 428 ± 66 ng/ml at 2h (p = 0.062) compared to pre-exercise. The 4h value of 409 ± 47 ng/ml also reached a statistical significant difference compared to the pre exercise value (p = 0.039). Thereafter, irisin values decreased, until 24h. The 2h and 6h irisin concentrations of STR were higher than CON (p = 0.039) and tended to be higher than CON (412 ± 54; p = 0.065), respectively. Time from end of training to mean of individual peak irisin concentration was not significantly different between END (47 ± 22 min after exercise) and STR (80 ± 30 min after exercise).

**Fig 3:**
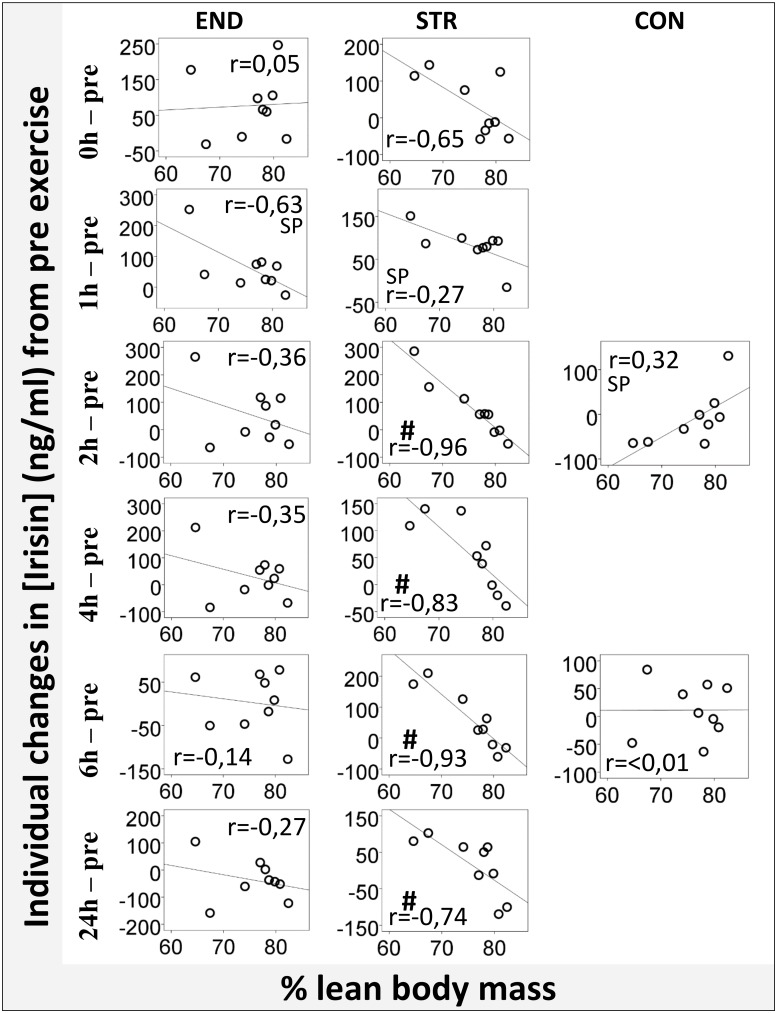
Lean body mass and exercise induced changes in irisin. Correlations between proportions of lean body mass and relative changes in plasma irisin following singular sessions of intense endurance exercise (END) or heavy strength training (STR), or on a control day (CON) without training. The X axis represent individual % lean body mass. The Y axis represent individual changes in irisin concentration from baseline to each of the different time points with blood samples. Correlation analyses were performed using Pearson’s method, except of the ones marked ^sp^, which were performed using Spearman’s method due to a lack of normal distribution. # = p≤0.05.

Following STR, individual training-induced changes in plasma irisin at 2h, 4h, 6h and 24h correlated very largely to nearly perfectly with % lean body mass (r = -0.74 to -0.96; Fig. 3). Similar but positive correlations were observed to % fat mass. This was not seen following END or CON. Training-induced changes in irisin did not correlate with BMI.

**Fig 4:**
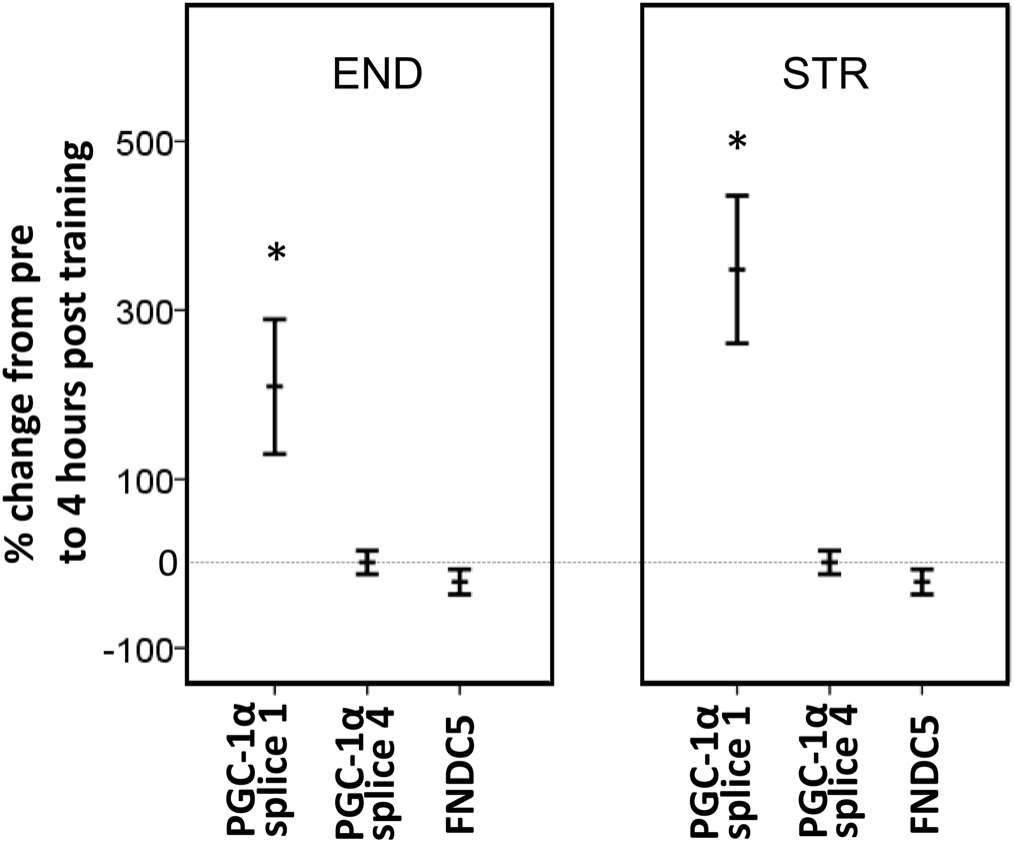
Changes in gene expression. Relative changes in gene expression of irisin-related genes following singular sessions of intense endurance exercise (END) or heavy strength training (STR). Muscle biopsies were sampled 4h post exercise. Data are mean values ± SEM. * = p≤0.05.

END and STR did not affect expression of FNDC5 in *musculus* vastus lateralis, measured 4h post-training (Fig. 4). Expression of PGC-1α splice variant 1 increased 2.1 ± 0.8—fold (p = 0.05) following END and 3.5 ± 0.9—fold (p = 0.01) following STR (Fig. 4). Exercise had no effect on PGC-1α splice 4 expression. There were no significant correlations between individual changes in PGC-1α splice 1 and changes in irisin concentrations from pre to post training samples.

## Discussion

This is the first study to show that irisin concentrations in blood increase in response to singular sessions of both high-intensity endurance exercise and heavy strength training in humans, using a highly standardized within-subject design. We provide a 24h time line for irisin after training, with seven sampling points, where others have typically limited their investigation to just a few points [20–27]. Peak concentrations of irisin occurred at 0h and 1h after training, supporting the notion that irisin is a training-inducible myokine [20–25], and that the increase is transient [21, 25, 37]. Previous studies have shown increased circulating irisin immediately or short time after endurance exercise [20–24]. Indeed, one study found circulating irisin levels to increase during the course of a 90 minutes treadmill exercise at 60% of VO_2max_, but at the end of exercise irisin levels had returned to baseline [37]. Altogether, it seems likely that irisin concentration in blood peaks within the first two hours following onset of exercise.

Observed increases in plasma irisin concentrations were not accompanied by increases in FNDC5 expression, measured 4h post-exercise. This suggests that regulation of irisin in blood does not rely on transcriptional regulation of FNDC5 in muscle in humans, at least not using the current timing of biopsy sampling. This is supported by data from other studies, who have found no or only minor effects of single training sessions on FNDC5 expression [26, 28]. The lack of changes in FNDC5 expression occurred despite 2–3 fold increases in PGC-1α splice 1 expression. A missing convergence between these two genes was also found by Rasche et al. [38], wherein electrical pulse stimulation of cultured human myotubes resulted in increased PGC-1α expression but not FNDC5 expression. It is possible that FNDC5 expression had increased and returned to baseline at a time-point offset from the timing of biopsy sampling employed in the present study.

Although the training-induced increases in irisin concentrations shown in the present study are supported by several studies [20–25], it contrasts findings of Pekkala et al. [26]. These discrepancies may be due to differences in exercise regimes [23, 25, 27]. Indeed, training intensity, but not age or fitness level seem to determine irisin secretion following endurance exercise [25]. It may also be due to methodological issues related to quantification of irisin [39].

To our knowledge, the present study is the first to link acute exercise-induced changes in irisin concentrations to body composition, evident as remarkably stable negative relationships with percentage lean body mass in STR. This suggests that subjects with the highest proportions of muscle mass entails the smallest increases in plasma irisin after strength training exercise. This relationship was present at all sampling points, except for the one exhibiting peak plasma irisin concentrations (1h). The mechanism behind this relationship may be related to muscle uptake of irisin from the blood or to decreased abilities of larger muscle masses to secrete irisin. Arguably, the latter scenario should have resulted in a similar significant relationship at peak irisin concentrations, which was not present. Interestingly, Ellefsen et al. [29] found a comparable negative correlation between changes in steady-state irisin levels after 12 weeks of heavy strength training, and lean body mass. The negative relationship between % lean body mass and post-exercise irisin concentration was not seen in CON or END groups.

Previously, the relationship between steady-state irisin levels and body composition has been explored in a few cross-sectional studies. For example, baseline irisin levels are low in amenorrheic athletes with low levels of body fat mass, compared to eumenorrheic athletes and non-athletes [40]. Irisin levels also seem to be positively correlated with biceps circumference (as an indicator of muscle mass) [41], and body weight, BMI and fat mass [18], and has been shown to decrease after weight loss caused by bariatric surgery [41].

The clinical relevance of irisin in humans was outside the scope of this study. This topic is disputed [4], and need to be further explored. Although a period of endurance [42] or strength training [29, 42] or both [21] does not increase steady-state irisin levels, there may certainly be cumulative health effects of transient training-induced increases in circulating irisin.

Our results, as well as previous studies [20–24, 37], suggest that future studies aiming to investigate effects of acute training sessions on irisin levels should sample blood within the first two hours of onset of exercise. The negative relationship between increases in irisin concentrations and proportions of lean body mass observed after strength training, but not after endurance training, calls for further attention. This may signify that different modes of exercise affect irisin biology in different manners.

## Conclusion

Our results supports the notion that irisin concentrations in blood increase transiently in response to single sessions of intense endurance exercise and heavy strength training in humans. Observed increases in plasma irisin concentrations were not accompanied by increases in FNDC5 expression, measured 4h post-exercise. Our results also suggest that individuals with low proportions of lean body mass exhibit more pronounced increases in irisin levels following resistance exercise than individuals with high proportion of lean body mass.
